# Cyclin-dependent kinase 11^p110^ (CDK11^p110^) is crucial for human breast cancer cell proliferation and growth

**DOI:** 10.1038/srep10433

**Published:** 2015-05-20

**Authors:** Yubing Zhou, Chao Han, Duolu Li, Zujiang Yu, Fengmei Li, Feng Li, Qi An, Huili Bai, Xiaojian Zhang, Zhenfeng Duan, Quancheng Kan

**Affiliations:** 1 Department of Pharmacy, The First Affiliated Hospital of Zhengzhou University, 1 Jianshe East Road, Zhengzhou 450052, China; 2 Sarcoma Molecular Biology Laboratory, Center for Sarcoma and Connective Tissue Oncology, Massachusetts General Hospital and Harvard Medical School, 55 Fruit Street, Boston, MA, USA; 3 Department of Infectious Diseases, The First Affiliated Hospital of Zhengzhou University, 1 Jianshe East Road, Zhengzhou 450052, China; 4 Department of Obstetrics and Gynecology, Zhengzhou Central Hospital Affiliated to Zhengzhou University, 195 Tongbai Road, Zhengzhou 450007, China; 5 Department of Pathology, Zhengzhou Central Hospital Affiliated to Zhengzhou University, 195 Tongbai Road, Zhengzhou 450007, China

## Abstract

Cyclin-dependent kinases (CDKs) play important roles in the development of many types of cancers by binding with their paired cyclins. However, the function of CDK11 larger protein isomer, CDK11^p110^, in the tumorigenesis of human breast cancer remains unclear. In the present study, we explored the effects and molecular mechanisms of CDK11^p110^ in the proliferation and growth of breast cancer cells by determining the expression of CDK11^p110^ in breast tumor tissues and examining the phenotypic changes of breast cancer cells after CDK11^p110^ knockdown. We found that CDK11^p110^ was highly expressed in breast tumor tissues and cell lines. Tissue microarray analysis showed that elevated CDK11^p110^ expression in breast cancer tissues significantly correlated with poor differentiation, and was also associated with advanced TNM stage and poor clinical prognosis for breast cancer patients. *In vitro* knockdown of CDK11^p110^ by siRNA significantly inhibited cell growth and migration, and dramatically induced apoptosis in breast cancer cells. Flow cytometry demonstrated that cells were markedly arrested in G1 phase of the cell cycle after CDK11^p110^ downregulation. These findings suggest that CDK11^p110^ is critical for the proliferation and growth of breast cancer cells, which highlights CDK11^p110^ may be a promising therapeutic target for the treatment of breast cancer.

Breast cancer is one of the most common cancers worldwide and the leading cause of cancer-related death in women^1^. Despite the development of potent cytotoxic, hormonal, and HER2-targeted agents for the treatment of breast cancer, the clinical outcome of patients remain unsatisfactory, and one third of women with localized disease will develop metastases and die of the disease^2^,^3^. While tumor-targeted agents have been extremely effective in treating HR+ and HER2+ breast cancers, *de novo* or acquired drug resistance is common and many cancers recur or progress^4^,^5^,^6^,^7^,^8^. Alternatively, triple-negative breast cancer (TNBC) does not yet have a clear tumor-specific receptor or pathway to target, and systemic therapy is restricted to cytotoxic chemotherapy^9^,^10^. Thus, identifying novel molecular targets and target-specific inhibitors against breast cancer is timely and essential.

It is evident that neoplastic cells display alterations in the progression of the normal cell cycle and abnormalities in the cell cycle are responsible for the majority of human neoplasias^11^,^12^. Cyclin-dependent kinases (CDKs) are a family of serine/threonine kinases, which are critical regulators of cell cycle progression and are constitutively expressed throughout the cell cycle^13^. CDKs are heterodimeric complexes composed of a catalytic kinase subunit and a regulatory cyclin subunit, regulated by their association with cyclins and endogenous inhibitors, as well as by positive phosphorylation and negative phosphorylation events^14^. In malignant cells, altered expression of CDKs and their modulators, including overexpression of cyclins and loss of expression of CDK inhibitors, result in deregulated CDK activity, providing a selective growth advantage. CDKs are often overexpressed and/or overactive in human cancers owing to various genetic and epigenetic events that affect their regulatory pathways, bringing about loss of checkpoint integrity, and ultimately resulting in uncontrolled cell proliferation^15^,^16^,^17^,^18^,^19^. Because of the critical roles in cell cycle progression, as well as the association of their activities with apoptotic pathways, CDKs and their associated pathways represent some of the most attractive targets for the development of anticancer therapeutics.

CDK11, formerly known as PITSLRE, is encoded by two highly homologous p34cdc2-related genes, *CDC2L1* and *CDC2L2* (Cell Division Control 2 Like) in humans. These two genes are localized in a genomic region that spans about 140 kb on human chromosome 1 band p36.3^20^. There is only one CDK11 gene, CDC2L1 in mouse. CDK11 involves three major isoforms, CDK11^p110^, CDK11^p58^, and CDK11^p46^, respectively^21^. The larger CDK11^p110^ protein kinase isoform is expressed in all human cancer cell lines examined so far, including the cell lines U-2OS, KHOS, Saos, Jurkat, Cem C7, HeLa, HEK 293, K562, HFF, and RNE^21^,^22^. The CDK11^p58^ protein is specifically translated from an internal ribosome entry site and expressed only in the G2/M phase of the cell cycle^23^. CDK11^p58^ detection depends primarily on the mitotic characteristics of a particular cell type. Although CDK11^p58^ shares the same sequences including the kinase domain as the C terminus of CDK11^p110^, the two isoforms possess different functions. CDK11^p58^ is closely related to cell cycle arrest and apoptosis in a kinase-dependent manner^24^,^25^,^26^. For human breast cancer, CDK11^p58^ has been identified as a negative regulator in the oncogenesis^27^,^28^. While the larger CDK11^p110^ isoform is mainly associated with transcription and RNA processes. Recently, CDK11^p110^ has been found to be critical for mesenchymal tissue-originated osteosarcoma cell growth and proliferation by a comprehensive human kinome-wide shRNA screening^22^. Moreover, similar effects of CDK11^p110^ on tumor cells have been confirmed in liposarcoma, which also arises from mesenchymal tissues^29^. However, the functional roles and molecular mechanisms of CDK11^p110^ in human breast cancer cell proliferation and growth are unknown.

In the present study, we explore the roles of CDK11^p110^ in the proliferation and survival of epithelial tissue-derived human breast cancer cells. Firstly, we detected CDK11^p110^ expression in a tissue microarray of human breast tumor samples and analyzed its correlation with the clinical characteristics of the patients. Additionally, we knocked down CDK11^p110^ expression with chemically synthetic small interfering RNA (siRNA) and examined the changes in human breast cancer cell proliferation, migration, apoptosis, and cell cycle. Our data show that CDK11^p110^ is highly expressed in human breast tumor cells, which correlates with poor prognosis for breast cancer patients. RNAi-mediated knockdown of CDK11^p110^ inhibits human breast cancer cell survival and proliferation *in vitro*. These results indicate that CDK11^p110^ plays an important role in the proliferation and growth of human breast cancer cells, and therefore warrants further evaluation as a therapeutic target of breast cancer.

## Results

### CDK11^p110^ is highly expressed in human breast cancer tissues and cell lines

**Figure 1:**
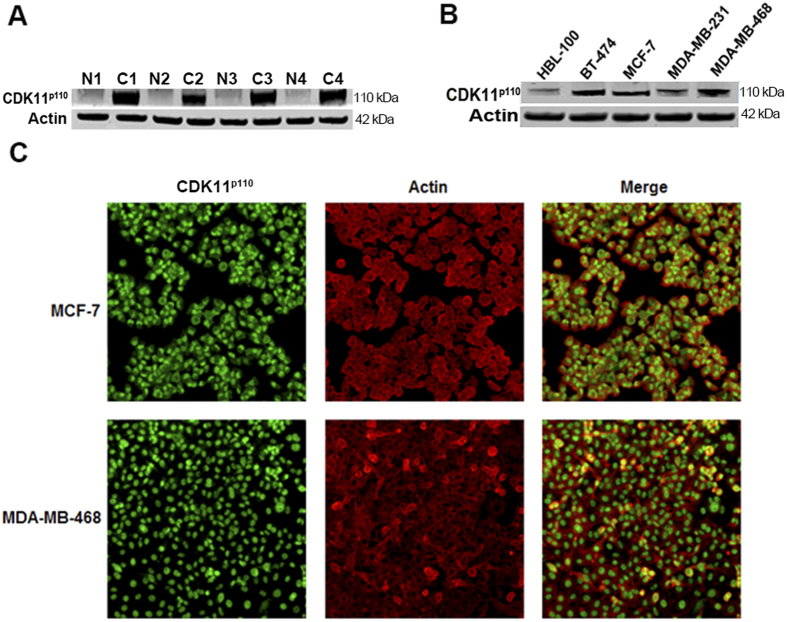
CDK11^p110^ is highly expressed in human breast cancer tissues and cell lines. (**A**) Breast tumor tissues and their adjacent normal tissues were lysed and immunoblotted to determine CDK11^p110^ expression, with Actin as an internal reference. All the gels were run under the same experimental conditions. Representative example of CDK11^p110^ expression in breast cancer tissues (C1-C4) and adjacent normal tissues (N1-N4) are shown. (**B**) Levels of CDK11^p110^ expression in normal human breast cell line HBL-100 and four breast cancer cell lines BT-474, MCF-7, MDA-MB-231, and MDA-MB-468 were detected using Western blot. All the gels were run under the same experimental conditions. (**C**) Expression of CDK11^p110^ in MCF-7 and MDA-MB-468 cells was assessed by immunofluorescence with antibodies to CDK11^p110^ and Actin. Cells were visualized under a fluorescence microscope after incubation with secondary fluorescent conjugated antibodies Alexa Fluor 488 goat anti-rabbit IgG (green) or Alexa Fluor 594 goat anti-mouse IgG (red).

To explore the potential roles of CDK11^p110^ in human breast cancer cell proliferation and growth, we first determined the expression of CDK11^p110^ in human breast tissues and cell lines. As demonstrated by Western blot, CDK11^p110^ was highly expressed in each of the tested human breast tumor tissues compared with the adjacent normal tissues (*P* < 0.01) (Fig. 1A, Supplementary Figure S1). Additionally, all four human breast cancer cell lines, with diverse histological staining characteristics (Supplementary Table S1), exhibited high levels of CDK11^p110^ expression, especially in BT-474, MCF-7, and MDA-MB-468 cells, whereas the expression of CDK11^p110^ was tightly regulated in the normal breast cell line (Fig. 1B). To further confirm the expression of CDK11^p110^ and determine its subcellular localization in breast cancer cell lines, immunofluorescence was performed in MCF-7 and MDA-MB-468 cells. As shown in Fig. 1C, CDK11^p110^ protein was mainly localized in the nucleus of breast cancer cells with some expression in the cytoplasm. For validation of CDK11^p110^ expression, we also determined CDK11^p110^ expression with a different commercially available CDK11^p110^ antibody from Cell Signaling Technology (CST, MA, USA catalog No: #5524) in the breast cancer tissues and adjacent normal tissues. Similar results of CDK11^p110^ expression were found in these samples (Supplementary Figure S1A).

### CDK11^p110^ expression levels correlate with the clinicopathological characteristics of breast cancer patients

**Figure 2:**
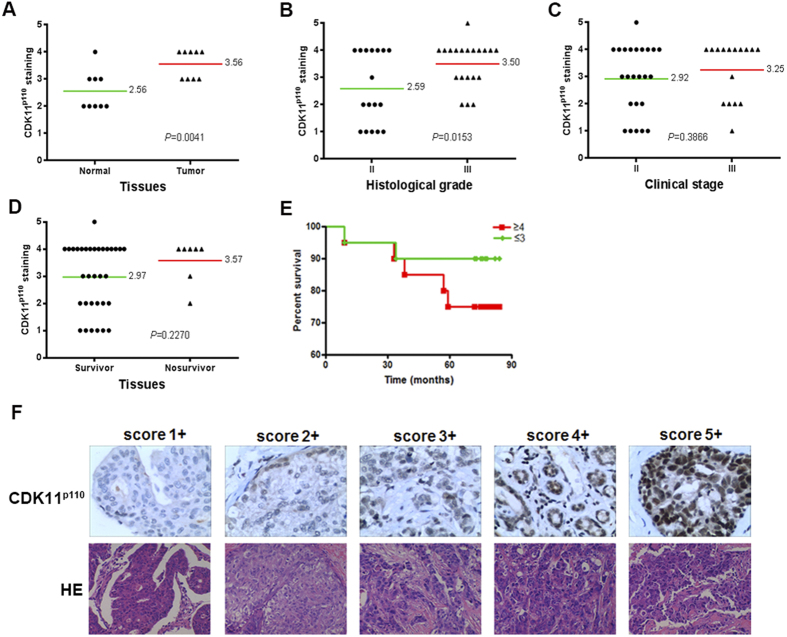
CDK11^p110^ expression levels correlate with clinicopathological characteristics of breast cancer patients. CDK11^p110^ levels in breast tumor tissue microarray were determined by immunohistochemistry, and the correlation of CDK11^p110^ expression with clinicopathological characteristics of breast cancer patients was evaluated. (**A**) Distribution of CDK11^p110^ staining scores among breast tumor tissues and their adjacent normal tissues. (**B**) Distribution of CDK11^p110^ staining scores among histological grade II and grade III breast tumor tissues. (**C**) Distribution of CDK11^p110^ staining scores among TNM II stage and III stage breast tumor tissues. (**D**) Distribution of CDK11^p110^ staining scores among survivor and nonsurvivor breast tumor tissues. (**E**) Kaplan-Meier survival curve of breast cancer patients with CDK11^p110^ low staining (CDK11^p110^ staining ≤3) or high staining (CDK11^p110^ staining ≥4). (F) Representative images of different immunohistochemical staining intensities of CDK11^p110^ in breast cancer tissues. On the basis of the percentage of cells with positive nuclear staining, CDK11^p110^ staining patterns were categorized into 6 groups: 0, no nuclear staining; 1+: < 10% positive cells; 2+, 10%-25% positive cells; 3+, 26%-50% positive cells; 4+, 51%-75% positive cells; 5+, > 75% positive cells. Original magnification ×400.

To further validate the clinical significance of CDK11^p110^ expression in patients with breast cancer, we detected CDK11^p110^ levels in a breast tumor tissue microarray by immunohistochemistry, and evaluated the correlation of CDK11^p110^ expression to the pathological characteristics and clinical prognosis of breast cancer patients. As illustrated in Fig. 2A, CDK11^p110^ expression levels were significantly higher in breast tumor tissues than that in their adjacent nontumorous tissues (*P* = 0.0041). Furthermore, elevated CDK11^p110^ expression in breast tumor tissues correlated with poor differentiation of tumor (*P* = 0.0153) (Fig. 2B). Additionally, CDK11^p110^ expression was upregulated in breast tumor tissues in advanced (III) TNM stage compared with that in primary (II) TNM stage, although there was no significant difference (*P* = 0.3866) (Fig. 2C). Based on data from over 72 months of follow-up, CDK11^p110^ expression levels in samples from nonsurvivors were higher than that from survivors, and more importantly, Kaplan–Meier survival analysis showed that the outcome for patients in the CDK11^p110^ high-staining (≥4) group was worse than for those in the CDK11^p110^ low-staining (≤3) group (Figs. 2D,E), although the difference was not statistically significant (*P* = 0.2270 and 0.2372, respectively).

### CDK11^p110^ knockdown inhibits human breast cancer cell proliferation ***in vitro***

**Figure 3:**
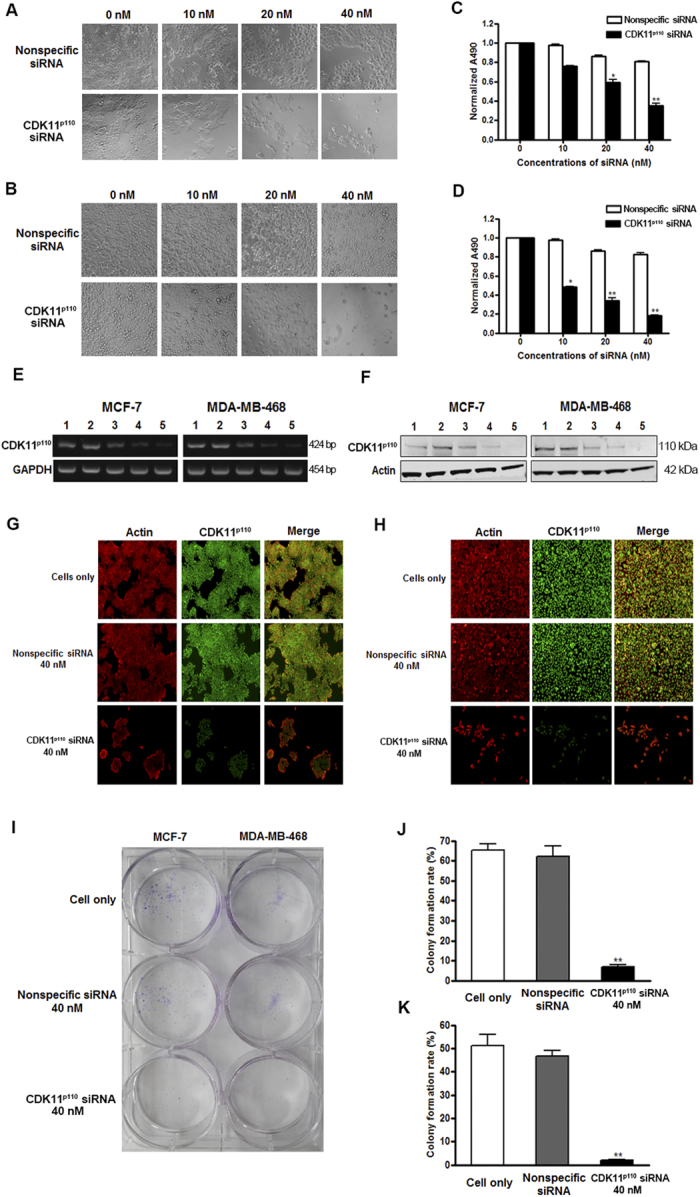
CDK11^p110^ knockdown inhibits human breast cancer cell proliferation *in vitro.* Human CDK11^p110^ siRNA were transfected into MCF-7 and MDA-MB-468 cells, followed by cell proliferation determination. (**A** and **B**) Morphological changes of MCF-7 and MDA-MB-468 cells, respectively, after CDK11^p110^ knockdown for 72 hours. (**C** and **D**) Cell viability changes determined by MTT assay of MCF-7 and MDA-MB-468 cells, respectively, after CDK11^p110^ knockdown for 72 hours. *P < 0.05, **P < 0.01 compared with the 0 nM siRNA group. (**E** and **F**) Downregulation of CDK11^p110^ mRNA and protein expression, determined by RT-PCR and Western blot, respectively, by CDK11^p110^ siRNA in MCF-7 and MDA-MB-468 cells. All the gels were run under the same experimental conditions. (1: 0 nM siRNA, 2: 40 nM nonspecific siRNA, 3: 10 nM CDK11^p110^ siRNA, 4: 20 nM CDK11^p110^ siRNA, 5: 40 nM CDK11^p110^ siRNA). (G and H) CDK11^p110^ knockdown induces cell death and decreases CDK11^p110^ expression in MCF-7 and MDA-MB-468 cells, respectively, detected by immunofluorescence. (**I**) Macroscopic images of cell colony formation alterations of MCF-7 and MDA-MB-468 cells, after CDK11^p110^ knockdown with 40 nM of CDK11^p110^ siRNA. (**J** and **K**) Cell colony formation rate changes of MCF-7 and MDA-MB-468 cells, respectively, after CDK11^p110^ knockdown with 40 nM of CDK11^p110^ siRNA. **P < 0.01 compared with the cell only group.

To further evaluate the functional role of CDK11^p110^ in human breast cancer cells proliferation *in vitro*, increasing concentrations of synthetic human CDK11^p110^ siRNA were transfected into MCF-7 and MDA-MB-468 cells. 72 hours later, the morphological changes were observed using phase contrast microscopy. As shown in Fig. 3A,B, CDK11^p110^ knockdown notably decreased the percentage of adherent cells, accompanied by dose-dependent death of transfected cells, which was not observed in the control or nonspecific siRNA transfected cells. Furthermore, the MTT assay also demonstrated that downregulation of CDK11^p110^ inhibited breast cancer cell viability in a dose-dependent manner (Figs. 3C,D). Additionally, the expression of CDK11^p110^ mRNA and protein in siRNA transfected cells was measured by RT-PCR and Western blot, respectively. As shown in Fig. 3, dose-dependent inhibition of CDK11^p110^ mRNA and protein expression were observed, which demonstrates that the suppressed cell viability was associated with the knockdown of CDK11^p110^ in breast cancer cells.

The role of CDK11^p110^ expression on breast cancer cell proliferation was further evaluated by immunofluorescence. Consistent with the results of the MTT assay, after transfection with 40 nM of CDK11^p110^ siRNA, MCF-7 and MDA-MB-468 cell viability decreased significantly, accompanied by reduced expression of CDK11^p110^ protein (Figs. 3G,H). As expected, there was no significant change in the cell proliferation and CDK11^p110^ expression in the breast cancer cells transfected with the same dose of nonspecific siRNA.

The alteration of human breast cancer cell proliferation after CDK11^p110^ knockdown was also assessed by a cell colony formation assay. As demonstrated in Fig, 40 nM of CDK11^p110^ siRNA transfection significantly inhibited the colony formation efficiency in both MCF-7 and MDA-MB-468 cells (both *P* < 0.01), which was not observed in cells transfected with the same dose of nonspecific siRNA.

### CDK11^p110^ knockdown suppresses human breast cancer cell migration

**Figure 4:**
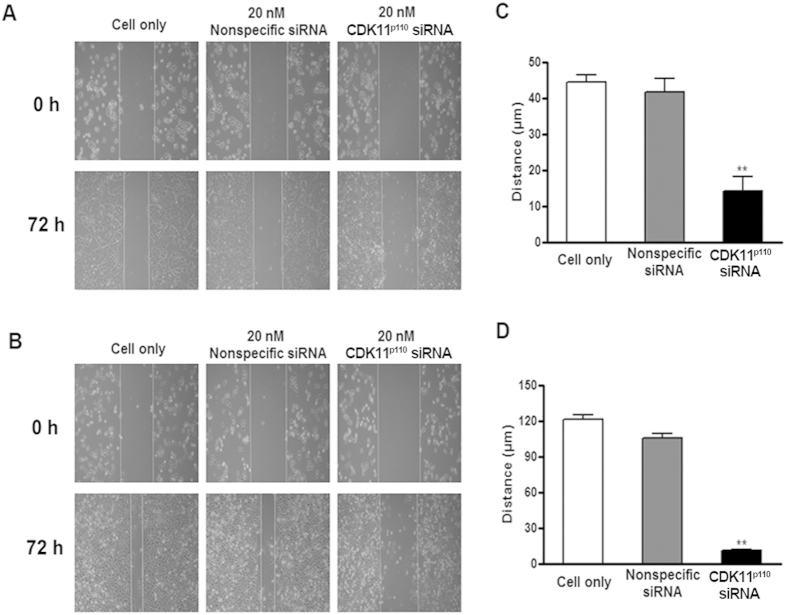
CDK11^p110^ knockdown suppresses human breast cancer cell migration. After transfection with 20 nM of CDK11^p110^ siRNA or nonspecific siRNA for 72 hours, the cell migration activity of MCF-7 and MDA-MB-468 cells was assessed by the wound healing assay. (A and B) Morphological images of MCF-7 and MDA-MB-468 cell migration, respectively, after CDK11^p110^ knockdown. (C and D) Migration distance of MCF-7 and MDA-MB-468 cells, respectively, after CDK11^p110^ knockdown. **P < 0.01 compared with the cell only group.

To investigate the effect of CDK11^p110^ knockdown on human breast cancer cell migration ability, a wound healing assay was performed after CDK11^p110^ siRNA transfection. Because 40 nM of CDK11^p110^ siRNA transfection resulted in pronounced cell death, we choose a siRNA dose of 20 nM for the wound healing assay. As illustrated in Fig. 4, after 72 hours of incubation, the migration activities of both MCF-7 and especially of MDA-MB-468 cells were significantly repressed in CDK11^p110^ siRNA transfected groups, as compared with nonspecific siRNA transfected and control groups (both *P* < 0.01).

### CDK11^p110^ knockdown induces cell apoptosis in human breast cancer cells

**Figure 5:**
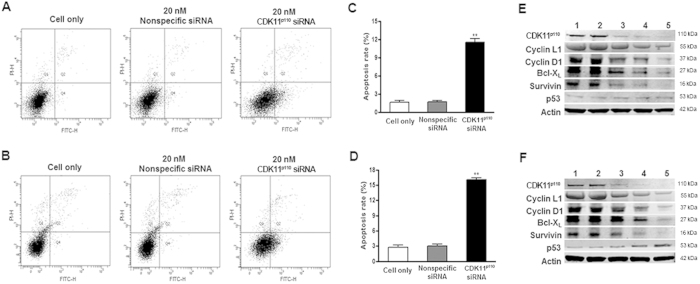
CDK11^p110^ knockdown induces cell apoptosis in human breast cancer cells. After transfection with 20 nM of CDK11^p110^ siRNA or nonspecific siRNA for 72 hours, the cell apoptosis of MCF-7 and MDA-MB-468 cells was assessed by flow cytometry analysis and Western blot. (**A** and **B**) Representative images of cell apoptosis alterations of MCF-7 and MDA-MB-468 cells, respectively, after CDK11^p110^ knockdown for 72 hours. (**C** and **D**) Apoptosis rate of MCF-7 and MDA-MB-468 cells, respectively, after CDK11^p110^ knockdown. **P < 0.01 compared with the cell only group. (E and F) Representative images of apoptosis-related proteins alterations in MCF-7 and MDA-MB-468 cells, respectively, after CDK11^p110^ knockdown for 72 hours. All the gels were run under the same experimental conditions. (1: 0 nM siRNA, 2: 40 nM nonspecific siRNA, 3: 10 nM CDK11^p110^ siRNA, 4: 20 nM CDK11^p110^ siRNA, 5: 40 nM CDK11^p110^ siRNA).

To explore the underlying mechanisms that inhibit breast cancer cell proliferation by CDK11^p110^ knockdown, we examined cell apoptosis using double independent experiments, including flow cytometry analysis and apoptosis-associated protein measurement. As demonstrated by flow cytometry analysis, increased apoptosis rates were observed in both MCF-7 and MDA-MB-468 cells transfected with 20 nM of CDK11^p110^ siRNA for 72 hours (Figs. 5A-D) (both *P* < 0.01). Meanwhile, as shown by Western blot, 72 hours of depletion of CDK11^p110^ by siRNA resulted in a dose-dependent decrease in the expression of anti-apoptotic proteins, including Survivin, Bcl-X_L_, cyclin D1, and CDK11^p110^-paired protein cyclin L1 in both MCF-7 and MDA-MB-468 cells. Furthermore, after CDK11^p110^ siRNA transfection, the quantity of p53 was dose-dependently increased in MDA-MB-468 cells, which express mutant p53, while the quantity of p53 protein remained at a barely detectable levels in MCF-7 cells with wild-type p53 (Figs. 5E,F).

### CDK11^p110^ knockdown induces human breast cancer cell cycle arrest in G1 phase

**Figure 6:**
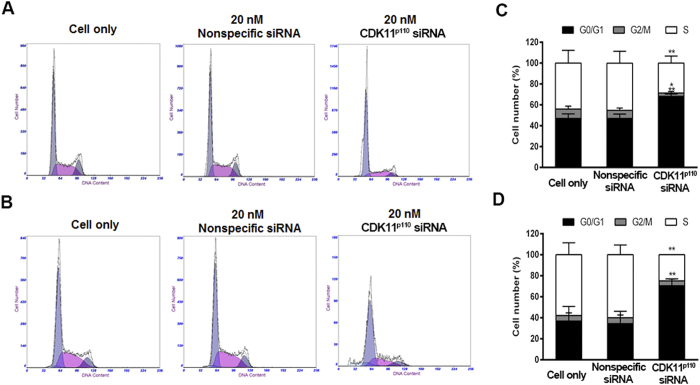
CDK11^p110^ knockdown induces human breast cancer cell cycle arrest in G1 phase. After transfection with 20 nM of CDK11^p110^ siRNA or nonspecific siRNA for 72 hours, the cell cycle distribution of MCF-7 and MDA-MB-468 cells was determined by flow cytometry. (**A** and **B**) Representative images of cell cycle distribution alterations of MCF-7 and MDA-MB-468 cells, respectively, after CDK11^p110^ knockdown. (**C** and **D**) Cell number in different cell cycle phases of MCF-7 and MDA-MB-468 cells, respectively, after CDK11^p110^ knockdown. *P < 0.05, **P < 0.01 compared with the cell only group.

To elucidate the potential mechanism underlying breast cancer cell apoptosis by CDK11^p110^ knockdown, flow cytometry analysis was used to determine cell cycle phase distributions in human breast cancer cells after CDK11^p110^ knockdown for 72 hours. As demonstrated in Fig. 6, after 20 nM of CDK11^p110^ siRNA transfection, a significant G1 cell cycle arrest accompanied by reductions in the fraction of cells in S phase was observed in both MCF-7 and MDA-MB-468 cells (both *P* < 0.01), suggesting that CDK11^p110^ knockdown was able to induce human breast cancer cell cycle arrest in G1 phase and inhibit DNA synthesis.

## Discussion

Cancer is characterized by uncontrolled proliferation and aberrant division of mammalian cells. CDKs and their related pathways control the cell cycle progression by maintaining ordered exit and entry to the different phases of the cell cycle. Aberrant expression or altered activity of CDKs results in escape of cells from the cell cycle control and leads to malignant transformation^12^,^14^,^15^,^30^,^31^,^32^,^33^. Therefore, inhibition of CDKs offers a promising therapeutic strategy in the defense against human malignancies.

The functions of CDK11 have been proved to be linked with the regulation of cell cycle, RNA transcription and processing, neuronal function, and apoptosis^21^,^34^,^35^,^36^,^37^. The potential for CDK11 to regulate these diverse cellular activities is unique in the CDK family and highlights that CDK11 may exert critical regulatory roles in human tumorigenesis, cancer cell growth and proliferation. These kinases have been renamed CDK11^p110^ and CDK11^p58^ when cyclins L1 and L2 were identified as regulatory subunits of CDK11^p110^. The smaller CDK11^p58^ isoform is expressed specifically in G2/M via an internal ribosome entry site (IRES) located within the CDK11^p110^ mRNA, and is closely related to cell cycle arrest and apoptosis upon binding to cyclin D3 partner^35^. In contrast, the larger CDK11^p110^ isoform, which is ubiquitously express throughout the cell cycle, is mainly associated with transcription and RNA processes by interacting with its regulatory partner cyclin L^21^,^38^,^39^,^40^,^41^,^42^,^43^,^44^. Moreover, both CDK11^p110^ and cyclin L1 proteins demonstrate increased expression in various cancers, and amplification of CCNL1 is associated with poor prognosis^45^,^46^,^47^. Recent research on mesenchymal tissue-originated tumors indicated that CDK11^p110^ plays important roles in osteosarcoma and liposarcoma cell survival^22^,^29^. However, the biological functions of CDK11^p110^ in human breast cancer cell growth and proliferation remain unclear. In the present study, we aimed to extend our study by investigating the roles of CDK11^p110^ in human breast cancer cell proliferation and growth. Our results demonstrated that CDK11^p110^ was highly expressed in human breast tumor tissues, which was significantly associated with poor differentiation, and also correlated with advanced TNM stage and poor clinical prognosis for breast cancer patients. *In vitro* knockdown of CDK11^p110^ expression by siRNA inhibited cell growth and migration, and induced apoptosis and cell cycle arrest in human breast cancer cells.

Firstly, we determined CDK11^p110^ expression in human breast tumor tissues and adjacent tissues, as well as in a normal human breast cell line and four breast cancer cell lines by Western blotting. The results indicated that CDK11^p110^ was highly expressed in human breast tumor tissues and cell lines. To further confirm the expression and subcellular localization of CDK11^p110^ in human breast cancer cells, immunofluorescence was performed on MCF-7 and MDA-MB-468 cells. The morphological images showed that CDK11^p110^ was highly expressed and mainly localized in the nucleus of both MCF-7 and MDA-MB-468 cells, with some cytoplasmic expression. To further validate the correlation of CDK11^p110^ expression with the pathological characteristics and clinical prognosis in breast cancer patients, we analyzed CDK11^p110^ protein levels by using a human breast tissue microarray. The results demonstrated that CDK11^p110^ expression levels are significantly higher in breast tumor tissues than that in their adjacent nontumorous tissues, Furthermore, CDK11^p110^ expression levels in breast tumor tissues positively correlated with the histological grade and clinical TNM stage of tumor. More importantly, CDK11^p110^ expression levels in samples from nonsurvivors were higher than that from survivors, and the outcome for patients in the CDK11^p110^ high-staining group was worse than for those in the CDK11^p110^ low-staining group, although there was no statistical significance. These data are likely confounded by the small, heterogeneous sample size, and larger studies with similar breast cancer patient samples are required. Nonetheless, these results provide a rationale for further evaluation of CDK11^p110^ as a marker for prognosis of breast cancer.

Recently, Chi and co-workers have evaluated the expression of a smaller CDK11 isoform, CDK11^p58^, in a breast tissue array by immunohistochemical staining^27^. The data showed that the disease-free survival (DFS) was significantly poorer in breast cancer patients with low CDK11^p58^ expression. Although CDK11^p58^ is structurally located within the C-terminal region of CDK11^p110^, their functions diverge distinctly due to their different crystal conformations^24^. For example, CDK11^p58^, in association with cyclin D3, was reported to negatively affect androgen receptor transcriptional activity, whereas CDK11^p110^ positively affected the transcription activity of androgen receptor^24^. Furthermore, it has been reported that treatment of the Fas-activated T cells with a serine protease inhibitor prevented apoptotic death and led to the accumulation of CDK11^p110^ isoform, but not the CDK11^p58^ isoform^48^. These discrepancies highlight that CDK11^p110^ and CDK11^p58^ may exert opposite functions on cancer cell growth and proliferation via different molecular mechanisms, which deserves further investigation.

To further characterize the functional roles of CDK11^p110^ in breast cancer cell survival and proliferation, we investigated the phenotypic alterations of MCF-7 and MDA-MB-468 cells after CDK11^p110^ knockdown by using chemically synthetic siRNA. The results showed that CDK11^p110^ knockdown inhibits human breast cancer cell proliferation in a dose-dependent manner accompanied by reduced expression of CDK11^p110^ mRNA and protein. Interestingly, after exposure to the same dose of CDK11^p110^ siRNA, MDA-MB-468 cell proliferation was repressed more significantly compared with MCF-7 cells, whereas the levels of downregulation of both CDK11^p110^ mRNA and protein had no significant difference. This phenotypic diversity may result from the heterogeneous genetic and biochemical background between MCF-7 and MDA-MB-468 cells, which changes the downstream signal transduction of CDK11^p110^. For example, MCF-7 cells express wild-type p53, while p53 in MDA-MB-468 cells is mutated^49^,^50^,^51^,^52^,^53^,^54^. Similarly, the wound healing assay illustrated that the migration activities of both MCF-7 and MDA-MB-468 cells were suppressed after CDK11^p110^ knockdown, with MDA-MB-468 cell migration more significantly inhibited. This altered phenotypic change may be caused by the intrinsic diversity in migration activity between these two breast cancer cell lines. Notably, MDA-MB-468 cells without CDK11^p110^ siRNA transfection migrated so quickly that they became confluent after 72 hours of incubation, while MCF-7 cells migrate slowly even without CDK11^p110^ knockdown.

To investigate how CDK11^p110^ sustains tumor cell survival and proliferation, we performed apoptotic assays after CDK11^p110^ knockdown in human breast cancer cell lines. As expected, cell apoptosis was observed in both MCF-7 and MDA-MB-468 cells after CDK11^p110^ siRNA transfection. The western blot assay for apoptosis-related proteins revealed that several anti-apoptotic proteins are reduced by CDK11^p110^ knockdown in breast cancer cell lines, suggesting that CDK11^p110^ is involved in cellular apoptotic signaling pathway. Thus, it can be reasoned that CDK11^p110^ knockdown inhibits human breast cancer cell proliferation by inducing cell apoptosis.

Considering that downregulation of CDK11^p110^ induces human breast cancer cell apoptosis leading to suppression of cell proliferation and that CDK11^p110^ exerts critical role in regulating cell cycle, we further explored the underlying mechanisms of cell apoptosis induction through CDK11^p110^ knockdown by determining alterations of the cell cycle. The results showed that cell cycle progression was blocked in G1 phase for both MCF-7 and MDA-MB-468 cells after CDK11^p110^ knockdown, with the MDA-MB-468 cell cycle arrested more significantly, which was consistent with the diversity in cell apoptosis induction and proliferation inhibition. Thus, we conclude that knockdown of CDK11^p110^ induces human breast cancer cell apoptosis via arresting cells in G1 phase of the cell cycle.

Cell cycle alterations are common in breast cancer. Newly popular targeted agent in breast cancer are CDK inhibitors which are agents inhibiting the function of different CDKs. CDK inhibitors have been tried as monotherapy and combination therapy in breast cancer clinical trials^55^,^56^. For example, CDK4 and CDK6 inhibitor palbocyclib is designed for a phase III trial for estrogen receptor (ER) positive breast cancer after showing favorable results in progression free survival in a phase II trial^57^. Flavopiridol, a pan-CDK inhibitor and targeting CDK2, CDK4, CDK6 and CDK9, synergizes with sorafenib to induce cytotoxicity and potentiate antitumorigenic activity in EGFR/HER-2 and mutant RAS/RAF breast cancer model systems^58^. In addition, CDK7/CDK9 inhibitors have also been evaluated in different stages of clinical trials in breast cancer. CDK7/9 inhibitor in ER-positive breast cancer cells has showed to prevent activating phosphorylation of ER-α^59^. More recent study revealed a synergistic effect existd between inhibitions of CDK 4/6 and PI3K in *PIK3CA* mutant breast cancer. CDK 4/6-PI3K inhibition is very effective in several *PIK3CA* mutant xenograft tumor models^60^. These ongoing clinical trials appeared promising and our current study suggests that CDK11^p110^ may be another potential therapeutic target for breast cancer.

Taken together, our current study demonstrated that CDK11^p110^ is highly expressed in human breast tumor tissues and cell lines, which correlates with the clinicopathological characteristics of breast cancer patients. *In vitro* knockdown of CDK11^p110^ by RNAi inhibits human breast cancer cell survival and proliferation by apoptosis induction via G1 cell cycle arrest. Our results suggest that CDK11^p110^ is critical for the proliferation and growth of human breast cancer cells, and may be a promising therapeutic target for the treatment of breast cancer patients.

## Methods

### Human breast tumor specimens

Eighteen pairs of breast tumor and their adjacent normal tissues were collected immediately after surgical resection at the First Affiliated Hospital of Zhengzhou University (Zhengzhou, China). No patients recruited in this study received any preoperative treatment. All diagnoses were confirmed histologically. Access to the materials was approved by the ethics committee of Zhengzhou University, Henan, China, and written informed consents were obtained from all patients. The collected tissues were frozen in liquid nitrogen until Western blot analysis for CDK11^p110^ expression levels. All experiments were performed in accordance with relevant guidelines and regulations.

### Tissue microarray and immunohistochemistry

A commercially available human tissue microarray, containing 40 breast cancer tumor tissues and 9 paired normal breast tissues, was purchased from Imgenex Corporation (Catalog number: IMH-364, CA, USA). Clinical data of patients for the microarray are detailed in Supplementary Table S2. CDK11^p110^ expression was examined with a SABC Immunohistochemistry Staining Kit (Boster, Wuhan, China) according to the manufacturer’s instructions. In brief, the paraffin-embedded slide was deparaffinized with xylenes and rehydrated with ethanol. After antigen retrieval in heated citrate buffer, the slide was incubated with 3% hydrogen peroxide solution to quench the endogenous peroxidase, followed by blocking with 5% BSA blocking buffer. Subsequently, the slide was incubated with rabbit polyclonal antibody to human CDK11^p110^ (SC-928, 1:50 dilution, Santa Cruz Biotechnology, CA, USA.) at 4 °C overnight, followed by incubation with biotin-conjugated secondary antibody at room temperature for 30 min. The slide was then incubated with Avidin-Biotin Complexes for 30 min, followed by coloration with DAB Chromogen Solution for 5 min. Finally, the tissue array was counterstained using hematoxylin (Boster, Wuhan, China), dehydrated in ethanol, and mounted with gelatine. The slide was imaged using a LeicaMicrosystems (Wetziar, Germany).

Immunostaining of the whole slide area was viewed and scored separately by three independent pathologists who were blinded to tumor characteristics and patient details of the samples. CDK11^p110^ staining patterns were categorized into 6 groups: 0, no positive staining; 1 +, <10% of positive cells; 2 +, 10%-25% positive cells; 3+, 26%-50% positive cells; 4 +, 51%-75% positive cells; and 5+, >75% of positive cells. Tumors with a staining score of ≥4 were defined as high expression and ≤3 were defined as low expression of CDK11^p110^.

### Cells culture and siRNA transfection

The normal human breast cell line HBL-100 and four breast cancer cell lines BT-474, MCF-7, MDA-MB-231, and MDA-MB-468 were purchased from the American Type Culture Collection (ATCC, Manassas, VA, USA). All five cell lines were cultured at 37 °C in a humidified 5% CO_2_ atmosphere in Dulbecco’s modified Eagle’s medium (DMEM) (Hyclone, USA) supplemented with 10% fetal bovine serum (GIBCO, USA), 100 units/ml penicillin G, and 100 μg/ml streptomycin. CDK11^p110^ knockdown in human breast cancer cells was performed by transfection of CDK11^p110^ siRNA synthesized by Shanghai GenePharma Co., Ltd (Shanghai, China). The siRNA sequence targeting CDK11^p110^ corresponded to coding regions (5’-AGAUCUACAUCGUGAUGAAtt-3’, antisense 5’-UUCAUCACGAUGUAGAU CUtg-3’) of the CDK11^p110^ gene. The nonspecific siRNA oligonucleotides (synthesized by Shanghai GenePharma Co., Ltd, Shanghai, China) were used as negative controls. MCF-7 or MDA-MB-468 cells were either plated on 96-well plates for cell proliferation assays or plated on 6-well plates for Western blot or Flow cytometry analysis. Various concentrations (0, 10, 20, and 40 nM) of CDK11^p110^ siRNA or nonspecific siRNA were transfected into MCF-7 or MDA-MB-468 cells with Lipofectamine RNAiMax reagent (Invitrogen, CA, USA) according to the manufacturer’s instructions. After 48 or 72 hours, transfected cells were subjected to subsequent analysis.

### Cell proliferation assay

72hours after CDK11^p110^ siRNA or nonspecific siRNA transfection, the morphological changes of MCF-7 or MDA-MB-468 cells were observed with a ZEISS microscope (Oberkochen, Germany). Meanwhile, *in vitro* cell viability of transfected cells was determined using the MTT assay. Briefly, at the end of CDK11^p110^ siRNA treatment, 20 μL of MTT (5 mg/mL, Sigma-Aldrich) was added to each well and the 96-well plates were incubated at 37 °C in a humidified 5% CO_2_ atmosphere for 4 hours. Finally, the resulting formazan product was dissolved with 150 μL of DMSO and the absorbance at a wavelength of 490 nm was measured on a Multiskan photometer microplate reader (Thermo Scientific, MA, USA).

### Immunofluorescence assay

MCF-7 or MDA-MB-468 cells were transfected with 40 nM of CDK11^p110^ siRNA or nonspecific siRNA in 8-well chambers (Thermo Scientific, NY, USA). After 48 hours, the cells were fixed with 4% paraformaldehyde (Solarbio, Beijing, China) for 15 min, followed by permeabilization with ice-cold methanol (Kermel, Tianjin, China) and blocked in 1% BSA (Solarbio, Beijing, China). The cells were then incubated with the CDK11^p110^ primary antibody (sc-928, Santa Cruz Biotechnology, 1:50 dilution) or β-Actin (sc-47778, Santa Cruz Biotechnology, 1:200 dilution) at 4 °C overnight, followed by incubation with Alexa Fluor 488 (Green) conjugated goat anti-rabbit antibody or Alexa Fluor 594 (red) goat anti-mouse antibody (Invitrogen, NY, USA) for one hour. Finally, cells were imaged on a ZEISS fluorescence microscope (Oberkochen, Germany) equipped with a Zen Imaging software.

### Cell colony formation assay

MCF-7 or MDA-MB-468 cells were transfected with 40 nM of CDK11^p110^ siRNA or nonspecific siRNA and seeded at a density of 100 cells per well in 6-well plates. After 10 days, when a macroscopic cell colony had formed, the cells were fixed in 4% paraformaldehyde (Solarbio, Beijing, China) and stained with Crystal Violet Staining Solution (Beyotime, Haimen, China), and the colonies were counted on a Zeiss microscope (Oberkochen, Germany) only if they contained more than 50 cells. The typical macroscopic images were photographed by a common Canon camera (Tokyo, Japan). Colony formation rates (%) were calculated using the following formula: (number of colonies / number of seeded cells) ×100%.

### Cell migration assay

Cell migration activity was detected by the wound healing assay. In brief, after transfection with 20 nM of CDK11^p110^ siRNA or nonspecific siRNA for 12 hours, the adherent MCF-7 or MDA-MB-468 cell layer was scraped in three parallel lines with a sterile 10 μL tip. After starved incubation with low-serum medium containing 3% FBS for an additional 72 hours, the cells were photographed under a Zeiss microscope (Oberkochen, Germany) equipped with a Zen Imaging software. The wound width was evaluated by measuring the distance between the two edges of the scratch at 5 sites in each image. Cell migration distance was determined using the following formula: (wound width at the starting time point - wound width at the end time point)/ 2.

### Semiquantitative reverse transcription-PCR

After 48 hours of CDK11^p110^ siRNA or nonspecific siRNA transfection, total RNA was extracted from MCF-7 and MDA-MB-468 cells with the TRIzol reagent (Invitrogen, USA). Reverse transcription of 100 ng of total RNA was performed using PrimeScript RT-PCR Kit (TaKaRa, Japan), and cDNA was subjected to PCR amplification. The sequences of CDK11^p110^ primers were CDK11-F: 5′-CGGGAAGTCAGAAATCGA-3′, and CDK11-R: 5′-CGTGGTGGTAAGGTGGA A-3′. As an internal reference, a GAPDH gene segment was amplified with primers GAPDH-F: 5′-GACCACAGTCCATGCCATCAC-3′ and GAPDH-R: 5′-GTCCACCA CCCTGTTGCTGTA-3′. Finally, the gene-specific PCR products were subjected to electrophoresis on 2% agarose gel and imaged using a BioSpectrum Imaging System (UVP, CA, USA) for CDK11^p110^ mRNA semiquantitative analysis.

### Protein preparation and Western blot

72 hours after CDK11^p110^ siRNA or nonspecific siRNA transfection, total protein was isolated from MCF-7 and MDA-MB-468 cells with RIPA Lysis Buffer (Beyotime, Haimen, China). The concentrations of the protein were quantified by BCA Protein Assay Reagents (Boster, Wuhan, China) with a NanoDrop spectrophotometer (Thermo Scientific, MA, USA). Western blotting was performed as previously described. Briefly, denatured proteins were run on an SDS-PAGE gel and then transferred to nitrocellulose membranes. After blocking in 5% nonfat milk for two hours, the membranes were incubated with rabbit polyclonal antibody to human CDK11^p110^ antibody 1 (Santa Cruz Biotechnology, cantalogy No: #sc-928, 1:500 dilution), CDK11^p110^ antibody 2 (Cell Signaling Technology, catalog No: #5524, 1:1000 dilution), cyclin L1 (sc-292385, Santa Cruz Biotechnology, 1:500 dilution), cyclin D1 (sc-753, Santa Cruz Biotechnology, 1:500 dilution), Survivin (sc-10811, Santa Cruz Biotechnology, 1:500 dilution), Bcl-X_L_ (sc-7195, Santa Cruz Biotechnology, 1:500 dilution), or mouse monoclonal antibody to human p53 (sc-126, Santa Cruz Biotechnology, 1:500 dilution), and β-Actin (sc-47778, Santa Cruz Biotechnology, 1:500 dilution) at 4 °C overnight. Following primary antibody incubation, the membranes were washed with TBST, and Goat anti-Rabbit IRDye 800CW (926-32211, 1:5000 dilution) or Goat anti-mouse IRDye 680LT secondary antibody (926-68020, 1:10000 dilution) (Li-COR Biosciences, NE, USA) was added, respectively. After incubation at room temperature for 2 hours, the bands were detected using Odyssey Infrared Fluorescent Western Blots Imaging System from Li-COR Bioscience (Lincoln, NE, USA). Quantification of Western blot results was analyzed with Odyssey software 3.0 (Li-COR Bioscience, Lincoln, Nebraska, USA).

### Flow cytometry analysis

After 72 hours of transfection with 20 nM of CDK11^p110^ siRNA or nonspecific siRNA, MCF-7 and MDA-MB-468 cells were subjected to flow cytometry analysis to examine the cell apoptosis and cell cycle changes. For cell apoptosis analysis, the cells were collected by trypsinization and resuspended in Binding Buffer, followed by staining with Annexin V-FITC and Propidium Iodide (KeyGEN BioTHCH, Nanjing, China) for 30 min, and then subjected to flow cytometry (FACSCantoll, BD, NJ, USA). For cell cycle analysis, the collected cells were fixed in 70% ethanol at 4 °C overnight, incubated in RNase A at 37 °C for 30 min, followed by dyeing with Propidium Iodide (KeyGEN BioTHCH, Nanjing, China) for 30 min. The DNA content was determined by flow cytometry (Beckman Coulter, CA, USA) and the population of cells in each cell cycle phase was analyzed by the equipped MultiCycle software (Phoenix Flow Systems, CA, USA).

### Statistical analysis

Statistical analysis was performed using the GraphPad PRISM 4 software (GraphPad Software, San Diego, CA, USA). Data are expressed as mean ± SD. Student’s t-test was used to determine the statistical significance of differences between groups. Survival analysis was performed using the Kaplan-Meier method, and significance was determined by the log-rank test. A *P* value of ≤ 0.05 was considered as statistically significant.

## Additional Information

**How to cite this article**: Zhou, Y. *et al.* Cyclin-dependent kinase 11^p110^ (CDK11^p110^) is crucial for human breast cancer cell proliferation and growth. *Sci. Rep.* **5**, 10433; doi: 10.1038/srep10433 (2015).

## Supplementary Material

Supplementary Information
