# Supplementary material for: Cyclin-dependent kinase 11p110 (CDK11p110) is crucial for human breast cancer cell proliferation and growth
Source: Sci Rep. 2015 May 20;5:10433. doi: 10.1038/srep10433 (PMC4438429; doi:10.1038/srep10433)
Supplement: Supplementary Information [file srep10433-s1.pdf]

Cyclin-dependent kinase 11^{p110} (CDK11^{p110}) is crucial for human breast cancer cell proliferation and growth

Yubing Zhou [a], Chao Han [a], Duolu Li [a], Zujiang Yu [c], Fengmei Li [d], Feng Li [a], Qi An [a], Huili Bai [e], Xiaojian Zhang [a], Zhenfeng Duan [a,b], Quancheng Kan [a*]

[a] Department of Pharmacy, The First Affiliated Hospital of Zhengzhou University, 1 Jianshe East Road, Zhengzhou 450052, China

[b] Sarcoma Molecular Biology Laboratory, Center for Sarcoma and Connective Tissue Oncology, Massachusetts General Hospital and Harvard Medical School, 55 Fruit Street, Boston, MA, USA

[c] Department of Infectious Diseases, The First Affiliated Hospital of Zhengzhou University, 1 Jianshe East Road, Zhengzhou 450052, China

[d] Department of Obstetrics and Gynecology, Zhengzhou Central Hospital of Zhengzhou University, 195 Tongbai Road, Zhengzhou 450007, China.

[e] Department of Pathology, Zhengzhou Central Hospital of Zhengzhou University, 195 Tongbai Road, Zhengzhou 450007, China.

[*]Corresponding author. Quancheng Kan, Department of Pharmacy, The First Affiliated Hospital of Zhengzhou University, 1 Jianshe East Road, Zhengzhou 450052, China

E-mail address: qckan19632012@163.com

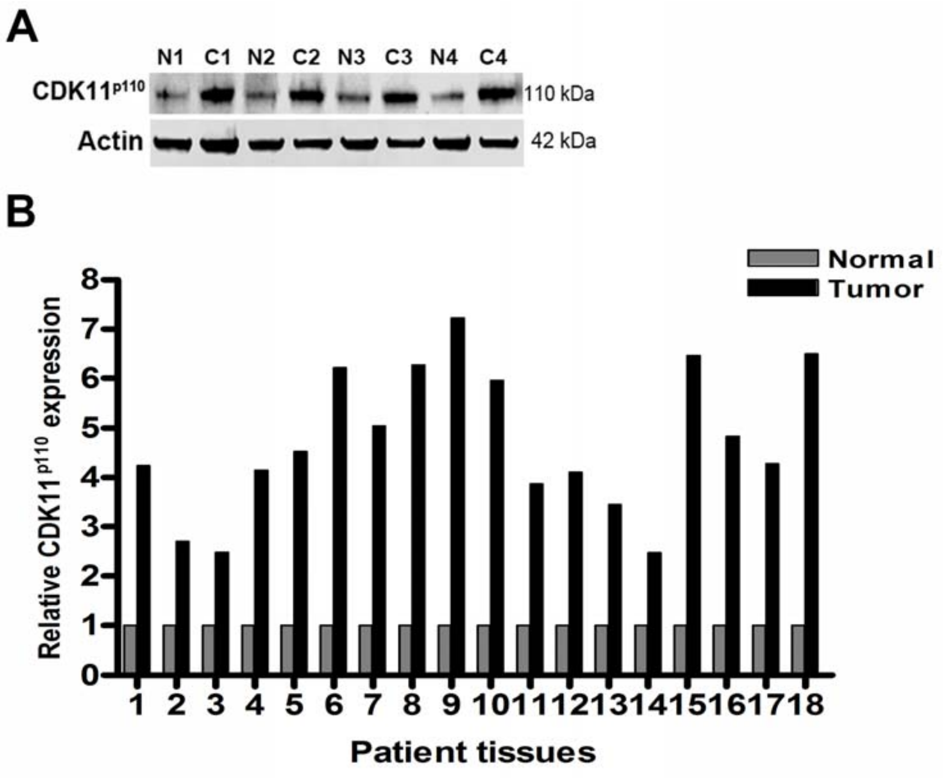

Supplementary Figure S1. Validation of CDK11^{p110} expression and relative expression levels of CDK11^{p110} in 18 breast tumor tissues

(A) Validation of CDK11^{p110} expression in breast tumor tissues and their adjacent normal tissues with CDK11^{p110} antibody from Cell Signaling Technology (CST, MA, USA). (B) Quantification of CDK11^{p110} expression in 18 pairs of breast tumor tissues and their adjacent normal tissues was determined by Western blot. The levels of CDK11^{p110} were quantified by the Odyssey software 3.0 (Li-COR Bioscience, Lincoln, Nebraska, USA) and normalized relative to the internal reference of Actin protein.

Supplementary Table S1 Molecular subtype and histological staining characteristics of the used human breast cancer cell lines

Cell lines	ER	PR	HER-2	EGFR	AR	CD5/6	Ki-67	p53	Subtype
**BT-474**	+	+	+	+	+	-	+	mutant	Luminal B
**MCF-7**	+	+	-	+	+	-	+	wild-type	Luminal A
**MDA-MB-231**	-	-	-	+	+	-	+	mutant	Basal
**MDA-MB-468**	-	-	-	+	+	-	+	mutant	Basal

*ER, estrogen receptor; PR, progesterone receptor; HER-2, human epidermal growth factor receptor-2; EGFR: epidermal growth factor receptor; AR, androgen receptor; "+", positive staining; "-", negative staining. The definition for each molecular subtype was based on the expression of ER, PR, HER2, EGFR and CK5/6.

Supplementary Table S2 Clinicopathological characteristics of breast cancer patients

No	Age (years)	Tumor Size (cm)	Clinical Stage	Histologic Grade	Tissue Status	Follow-up Months	Follow-up Results
1	59	3.0	II A	II	Tumor	84	Alive
2	48	3.5	II A	II	Tumor	84	Alive
3	42	4.2	III A	II	Tumor	84	Alive
4	37	3.0	III A	III	Tumor	34	Dead
5	37	2.5	II A	III	Tumor	84	Alive
6	55	2.5	II A	II	Tumor	83	Alive
7	55	10.0	III C	II	Tumor	57	Dead
8	36	6.0	II B	II	Tumor	82	Alive
9	52	2.5	III A	III	Tumor	82	Alive
10	40	4.5	II A	II	Tumor	82	Alive
11	51	2.5	II A	III	Tumor	82	Alive
12	55	3.0	II A	III	Tumor	80	Alive
13	60	2.5	II A	III	Tumor	79	Alive
14	45	5.0	II B	III	Tumor	38	Dead
15	38	5.2	III A	II	Tumor	79	Alive
16	53	2.2	II B	II	Tumor	78	Alive
17	48	2.5	II B	II	Tumor	78	Alive
18	46	4.5	II A	UK	Tumor	77	Alive
19	40	5.5	II B	III	Tumor	77	Alive
20	51	2.5	II B	UK	Tumor	77	Alive
21	56	4.5	II A	III	Tumor	77	Alive
22	45	3.5	II A	UK	Tumor	77	Alive
23	42	3.0	III C	II	Tumor	76	Alive
24	47	3.0	II B	II	Tumor	76	Alive
25	39	2.5	II B	III	Tumor	76	Alive
26	51	4.0	II B	III	Tumor	76	Alive
27	49	2.5	II A	III	Tumor	78	Alive
28	57	5.5	III A	II	Tumor	75	Alive
29	52	5.0	II B	II	Tumor	75	Alive
30	41	10.0	III A	II	Tumor	59	Dead
31	48	3.5	III C	III	Tumor	9	Dead
32	34	3.0	II B	III	Tumor	82	Alive
33	37	2.5	III C	III	Tumor	81	Alive
34	58	3.5	III C	III	Tumor	9	Dead
35	37	4.5	III C	III	Tumor	78	Alive
36	66	5.0	III A	III	Tumor	75	Alive
37	51	3.0	II B	III	Tumor	73	Alive
38	41	3.5	III C	III	Tumor	72	Alive
39	56	6.0	III A	II	Tumor	72	Alive
40	47	10.0	III C	II	Tumor	33	Dead

41	37	/	/	/	Normal (match to 5#)	84	Alive
42	48	/	/	/	Normal (match to 31#)	9	Dead
43	36	/	/	/	Normal (match to 8#)	82	Alive
44	40	/	/	/	Normal (match to 10#)	82	Alive
45	51	/	/	/	Normal (match to 11#)	82	Alive
46	55	/	/	/	Normal (match to 12#)	80	Alive
47	60	/	/	/	Normal (match to 13#)	79	Alive
48	58	/	/	/	Normal (match to 34#)	9	Dead
49	37	/	/	/	Normal (match to 35#)	78	Alive

*UK, unknown
